# Mr. Weaver, on Apoplexia Hydrocephalica

**Published:** 1806-10

**Authors:** W. Weaver

**Affiliations:** Surgeon. Walsall


### *To the Editors of the Medical and Physical Journal.*

Gentlemen,

Among the various privileges which we enjoy, few stand more conspicuous than that of having an opportunity of holding literary intercourse with gentlemen, whose principles are actuated by the dictates of reason, supported upon the permanent basis of temperance, coolness, and moderation, "influenced by a laudable zeal for the alleviation of human misery;" such motives are well calculated to promote our mutual happiness, the prosperity of science, and the cultivation of genius. Ere I proceed, I must candidly acknowledge, I am no great advocate for enlisting under the Banners of Medical Controversy; which, which, to the disgrace of a few, has greatly exceeded the bounds of decency and moderation: supported by much virulence and personal invective, such conduct is ill adapted to inspire us with confidence, or contribute to our scanty store of medical knowledge. But as the motives of C. S. are those of "philanthropy," and as he politely requests an elucidation of my case of "Apoplexia Hydrocephalica," I will endeavour to convince him of the propriety of the mode of "treatment," as well as the "position" laid down; previous to which, permit me to acknowledge the obligation I am under for the seeming good opinion he has imbibed of an unknown individual, (probably premature). Tho' he bows assent to the "Preliminary Observations," yet his superior judgment revolts at the mode of "treatment," and "position," or principle inferred, notwithstanding the successful termination of the case, which I conceive sufficient to remove all prejudice. It appears, he disallows that the disease is capable of being cured if taken in time, prior to the accession of the second stage; "although, (he says) I think, by due attention and acumen in the practitioner, in ascertaining the disease at his first attendance, and by active means then zealously employed, it may very often be arrested in its progress; but, if that is to be the case, if its gigantic strides are to be impeded, it must be accomplished by different and more active means than those employed by Mr. W." But I will venture to affirm, however gigantic or Herculean his ideas may be, from indubitable authority, independent of my own experience, that instances have occurred, in which a cure has been effected, and not by "means different or more active" than those used in the present case; at the same time, I deny the possibility of our being always capable of "ascertaining" the reality of "the disease," or discriminating it from others, productive of similar symptoms, "at the first attendance," which was before observed, "as irritation occasioned by worms, dentition, crudities in the stomach, &c."

In the sequel of his Strictures, he wishes to be informed, if, "at my first visit, there appeared no flushed face, no contracted pupil, and fever." The two former were not obvious, the latter evident. The case is, as was represented, without intentionally adding or diminishing; from which representation, it appeared doubtful whether the then existing symptoms were the effects of causes above related, unconnected with hydrocephalus, which, in the first instance, led to the means used, as an Emetic, &c.; which which would not have been had recourse to, had not the symptoms indicated. The operation of an emetic, I was well satisfied, could not in any respect be productive of pernicious tendency, or "add fuel to the fire." On the contrary, I have reason to believe, that by its action on the absorbent system, by exciting a determination to the skin, by evacuating the stomach, and, probably, by removing any thing that might (by its gravity on the stomach) press upon the Aorta, prevent that which he was apprehensive it would occasion, — a "determination of blood to the Head." Its utility in hydrocephalus, as well as in serous apoplexy, more than counterbalances its supposed ill effects.

Admitting an emetic was not proper in hydrocephalus, (which it appears I do not accede to) yet I must again observe, the disease, "at the first visit," was not "ascertained;" and as the child had aforetime voided worms per os, which were discovered by the father, I had no reason to doubt their existence. As such, an emetic was no deviation from a regular mode of practice. On my second visit, it appears, the disease had assumed its regular form, and the "means used were sufficiently active to arrest it in its progress;" and not only capable of "impeding its gigantic strides," but of conquering the enemy.

I am astonished that C. S. should, in one part, ridicule the insignificancy of "the *means used*," and in another, express his astonishment "that such very powerful drugs should have been exhibited to the extent" mentioned. —Why should C. S. feel so much surprize at a child three years old taking in the course of five days, pulv. digital. gr. viij, and calomel gr. xvj? He would probably have had some reason for being alarmed, if it had taken the quantity in one day, or at one dose. When we reflect how much more difficult it is to excite the absorbent system of a child into action, than that of the adult, we need not express sentiments of wonder at the "means" used, or at the extent to which they were carried; suffice to repeat, they had the happy tendency not only of "improving," but establishing "health and strength;"— the child still lives, as a shining meteor, to corroborate the fact. If C. S. had perused the case with that attention required, it would have discovered to him a mode of treatment the nature of the disease required, strictly "antiphlogistic," (a term now almost exploded) viz. topical bleeding, blisters, medicines "debilitating" (and

"sedative" if he prefers the term, though its propriety is doubted.)

As to the virtues of the digitalis, I can speak with some degree of positive assurance, and, I trust, with confidence; it was gathered by myself in the season of the year recommended by the late Dr. Withering, and carefully dried and preserved according to his directions. In order to obviate doubts and fears, I have only to observe, that should I again have the same disease to combat with, I should not hesitate in using the same means, and, I hope, with the like good effects; at the same time, were the bowels to be equally constipated, and the absorbent system difficult to be acted upon, I might not only be inclined to increase the quantity of calomel, but the digitalis also. I am, &c.

*Walsall, Aug.* 4, 1806. W. WEAVER, Surgeon.

